# Archaeal and bacterial communities in three alkaline hot springs in Heart Lake Geyser Basin, Yellowstone National Park

**DOI:** 10.3389/fmicb.2013.00330

**Published:** 2013-11-12

**Authors:** Kara Bowen De León, Robin Gerlach, Brent M. Peyton, Matthew W. Fields

**Affiliations:** ^1^Department of Microbiology, Montana State UniversityBozeman, MT, USA; ^2^Center for Biofilm Engineering, Montana State UniversityBozeman, MT, USA; ^3^Thermal Biology Institute, Montana State UniversityBozeman, MT, USA; ^4^Department of Chemical and Biological Engineering, Montana State UniversityBozeman, MT, USA; ^5^National Center for Genome ResourcesSanta Fe, NM, USA

**Keywords:** 16S rRNA pyrosequencing, alkaline hot spring, Heart Lake Geyser Basin, methanogenic community, phylogeny, *Thermus*, Yellowstone National Park, thermoalkaline

## Abstract

The Heart Lake Geyser Basin (HLGB) is remotely located at the base of Mount Sheridan in southern Yellowstone National Park (YNP), Wyoming, USA and is situated along Witch Creek and the northwestern shore of Heart Lake. Likely because of its location, little is known about the microbial community structure of springs in the HLGB. Bacterial and archaeal populations were monitored via small subunit (SSU) rRNA gene pyrosequencing over 3 years in 3 alkaline (pH 8.5) hot springs with varying temperatures (44°C, 63°C, 75°C). The bacterial populations were generally stable over time, but varied by temperature. The dominant bacterial community changed from moderately thermophilic and photosynthetic members (*Cyanobacteria* and *Chloroflexi*) at 44°C to a mixed photosynthetic and thermophilic community (*Deinococcus-Thermus*) at 63°C and a non-photosynthetic thermophilic community at 75°C. The archaeal community was more variable across time and was predominantly a methanogenic community in the 44 and 63°C springs and a thermophilic community in the 75°C spring. The 75°C spring demonstrated large shifts in the archaeal populations and was predominantly *Candidatus Nitrosocaldus*, an ammonia-oxidizing crenarchaeote, in the 2007 sample, and almost exclusively *Thermofilum* or *Candidatus Caldiarchaeum* in the 2009 sample, depending on SSU rRNA gene region examined. The majority of sequences were dissimilar (≥10% different) to any known organisms suggesting that HLGB possesses numerous new phylogenetic groups that warrant cultivation efforts.

## Introduction

The Heart Lake Geyser Basin (HLGB) is located along Witch Creek and the northwestern shore of Heart Lake in Yellowstone National Park (YNP). The basin lies at the intersection of the Yellowstone Caldera and a fault line along the east side of the Red Mountains and Mount Sheridan. Hot springs along the creek have unique pH conditions that change from acidic to alkaline as the creek nears the lake and have geochemistries characteristic of acid and neutral Cl-rich waters (i.e., elevated Na, Cl, and SiO_2_) (Lowenstern et al., 2012). Likely due to the remote nature of the HLGB, little is known about the populations in these springs. In a survey of green non-sulfur mat communities throughout YNP, a HLGB spring (pH 8.7, 47–50°C) was included in the analysis and was determined to be exclusively *Roseiflexus* (Boomer et al., 2002). Furthermore, ammonia-oxidizing enrichments from Heart Lake resulted in the cultivation of a novel archaeon *Candidatus Nitrosocaldus yellowstonii* (de la Torre et al., 2008). Both of these studies targeted specific physiologies and did not analyze the community. The purpose of the present study was to better understand the communities in this remote area of YNP by elucidating the diversity and stability of bacterial and archaeal populations in alkaline springs of different temperatures.

## Materials and methods

### Site description and sample preparation

**Figure 1:**
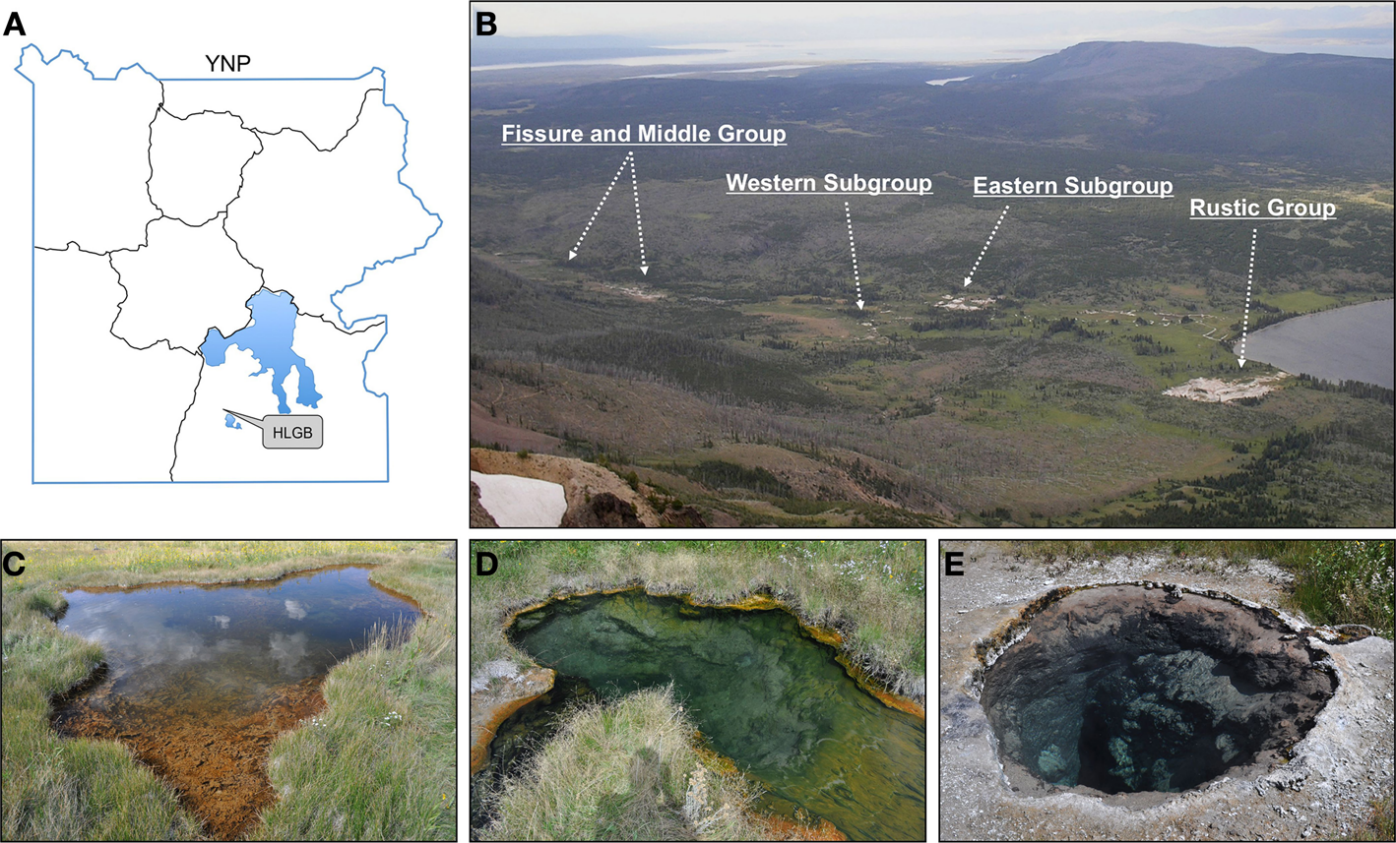
**Heart Lake Geyser Basin and the 3 springs selected for analysis.** The HLGB (as viewed from Mount Sheridan; **B**) is located in the southern portion of YNP **(A)**. The 3 springs selected were located in the Western Subgroup. The springs were at 44°C **(C)**, 63°C **(D)**, and 75°C **(E)** and all were at pH 8.5 in 2007. The image of the 44°C spring **(C)** was taken in 2009 and shows tears throughout the microbial mat that were not observed in 2007 or 2008.

Three springs were selected in the Western subgroup of the Lower HLGB with approximately the same pH (pH 8.5), but varying temperature from 44 to 75°C (Figure 1). The 44°C spring (44.29047 N, 110.50998 W) was ~5 m wide by 1 m deep (measured near an abrupt edge) with a steeply sloping edge, little or no thermal gradient, and contained a thick (1–2 cm) red and green microbial mat. The effluent of the spring flowed into a small stream consisting of effluent from distal springs, which combines with a spring at 63°C (44.29068 N, 110.50983 W) at a bend in the stream, forming an eddy ~1.5 m wide by 1 m deep. Near the effluent of the 63°C spring, a thin, green microbial mat covered a thick, loose black layer of organic matter. A third spring at 75°C (44.29047 N, 110.50987 W), in close proximity to these springs but not connected by surface hydrology, was ~2 m wide, >4 m deep, with an abrupt edge and no visible biomass. The 44, 63, and 75°C springs were identified as HLW028, HLW004, and HLW009, respectively, from the YNP Thermal Inventory according to GPS coordinates and photographs. The YNP Thermal Inventory was provided by the YNP Spatial Analysis Center and made available by the National Science Foundation YNP Research Coordination Network (www.rcn.montana.edu).

Samples were collected in July 2007, September 2008, and August 2009. The 44 and 63°C spring samples contained mat material and surrounding water. The 75°C spring sample consisted of water and sediment. The water/sediment and water/mat slurry samples were collected in conical vials (50 mL volume) for each spring, immediately placed on dry ice, and stored at −80°C until DNA extraction. The combined samples were used to capture as much diversity as possible from the respective springs, and future studies plan to target community structure along the boundaries of bulk phase and biofilm. After centrifugation at 3900 ×g for 20 min, ~5 g was used for extraction via a protocol modified from Zhou et al. (1996) that has been described previously (Bowen De León et al., 2012). DNA extracts from the 75°C spring were concentrated 20-fold using the Wizard^®^ SV Gel and PCR Clean-Up System (Promega Corporation, Madison, WI, USA) according to the manufacturer's protocol. DNA was quantified with the dsDNA BR assay on a Qubit^®^ 1.0 fluorometer (Invitrogen, Carlsbad, CA, USA).

### Aqueous geochemical analysis

In 2009, prior to slurry collection, aqueous samples were collected at each spring and filtered (0.2 μm nylon syringe filter) on site into a 50 mL conical vial. An aliquot of each filtered aqueous sample was diluted 1:100 in 5% trace metal grade nitric acid for preservation. Dissolved Na, Mg, K, Ca, Al, As, Fe, and Be, and trace elements V, Cr, Mn, Co, Ni, Cu, Zn, Se, Mo, Ag, Cd, Sb, Ba, Tl, Pb, Th, and U were analyzed in the acidified sample on an Agilent 7500 inductively coupled plasma mass spectrometer (ICP-MS; Agilent 7500ce ORS, Foster City, CA, USA) in Montana State University's Environmental and Biofilm Mass Spectrometry Facility. Helium was used as a collision gas to reduce interferences of argon oxide ions. Predominant inorganic anions (F^−^, Cl^−^, and SO^2−^_4_) were analyzed in the non-acidified sample via ion chromatography (IC) as previously described (Inskeep et al., 2005). Samples collected in closed headspace serum bottles were used for total carbon (TC), non-purgable organic carbon (NPOC), and total nitrogen (TN) measurements with a Shimadzu TOC-V_CSH_ with attached TNM-1 (Shimadzu Scientific Instruments, Columbia, MD). Total dissolved sulfide (DS) was analyzed on an unfiltered sample using the amine sulfuric acid method (APHA, 1998).

### DNA sequencing

Bacterial small subunit (SSU) rRNA gene amplification was performed using universal bacterial primers 530F (5′-GTGCCAGCMGCNGCGG-3′) and 1100R (5′-GGGTTNCGNTCGTTR-3′) under conditions described in Hwang et al. (2009). Archaeal primers 751F (5′-CCGACGGTGAGRGRYGAA-3′) and 1204R (5′-TTMGGGGCATRCNKACCT-3′) (Baker et al., 2003) were used to amplify the archaeal population with the following PCR protocol: 94°C for 5 min; cycling 94°C for 30 s, 55°C for 30 s, and 72°C for 2 min; followed by 72°C for 7 min and 4°C hold. All primers had 10-nt barcodes on the 5′-end for sample identification purposes. Cycle numbers were optimized for each sample to minimize PCR-induced artifacts (Acinas et al., 2005; Sipos et al., 2007; Wu et al., 2010) with 10–12 ng DNA per reaction (5 μL concentrated DNA for the 75°C spring samples for which the DNA concentration remained below the detection limit of 1 ng/μL). PCR was repeated with sufficient reactions to yield 500 ng for FLX pyrosequencing or 50 ng for FLX-Junior pyrosequencing (see below). PCR products were excised and extracted from a 0.8% agarose gel using an Ultrafree^®^ -DA gel extraction column (Millipore Corporation, Bedford, MA, USA), concentrated with the Wizard^®^ kit (above), and quantified with the dsDNA BR assay on a Qubit^®^ 1.0 fluorometer (above).

Pyrosequencing for the 2007 bacterial amplicons was done with GS-FLX pyrosequencing technology at the Medical Biofilm Research Institute (Lubbock, TX, USA). The archaeal 2007 amplicons and the 2008 bacterial and archaeal amplicons were sequenced on a 454 GS-FLX Titanium™ Pyrosequencer (454 Life Sciences, Branford, CT, USA) at SeqWright, Incorporated (Houston, TX, USA). The 2009 amplicons were prepared and sequenced on an in-house GS Junior Titanium™ Pyrosequencer as follows. PCR products were combined for 4–6 samples at 50 ng/sample and were concentrated using Agencourt^®^ AMPure^®^ XP beads (Beckman Coulter, Inc., Brea, CA, USA) per Roche 454 Life Sciences (Branford, CT, USA) protocol in Amplicon Library Preparation Method Manual section 3.2.2 (Rev. June 2010) with the following modifications: AMPure^®^ XP beads were added at 1.8 times the sample volume and the DNA was re-suspended in 16 μL TE buffer. Roche 454 Lib-L adaptors were ligated per Roche Rapid Library Preparation Method Manual (Rev. June 2010) beginning at section 3.2. Emulsion PCR (emPCR) was prepared at 0.5 DNA molecules/bead per Roche protocol with the following modifications: emPCR reagents were optimized for our sequence length (355 μL water, 515 μL additive, 300 μL AmpMix, 105 μL AmpPrimer, 70 μL Enzyme Mix, 2 μL PPiase) and emPCR conditions included an initial melting time at 94°C for 4 min followed by 50 cycles of 94°C for 30 s and 60°C for 10 min and held at 10°C until bead preparation per Roche Technical Bulletin No. 2011-001. Bead preparation and sequencing on a GS Junior Titanium pyrosequencer were done according to Roche protocol. Sequences were submitted to the NCBI Short Read Archive under the BioProject accession number PRJNA207095.

### Sequence analysis

The Titanium pyrosequences were trimmed and refined by primer error, ambiguous nucleotides (Ns), length, and quality score as described previously (Bowen De León et al., 2012). Bacterial and archaeal sequences were analyzed by quality score using quality-score (Q) cutoffs of Q30 and Q32 for the forward and reverse regions, respectively, with 10 or 15% of nucleotides allowed to be below the cutoff for the respective region. The 2007 FLX pyrosequences were refined by primer error, Ns, and length as first described in Huse et al. (2007). Because the V6 region (1100R) of the bacterial SSU rRNA gene has been shown to yield poor recovery for identification and sample clustering (Liu et al., 2007; Claesson et al., 2009; Claesson and O'Toole, 2010), the V6 OTUs were compared in Supplementary Figure 1. Furthermore, less is known regarding the ability of short archaeal SSU rRNA gene sequences to make correct identifications; thus, both forward and reverse reads were analyzed for comparison.

Sequences were analyzed for chimeras via ChimeraSlayer (Haas et al., 2011). Sequences identified as chimeric were removed with the exception of those called chimeric by ChimeraSlayer at the intra-genus level, which were not considered chimeric and were not removed from the dataset. Non-chimeric sequences were aligned, clustered at 97%, and a representative sequence was selected for each cluster using the Ribosomal Database Project's (RDP) Pyrosequencing Pipeline (Cole et al., 2009). Sequences were compared against the NCBI nucleotide database and the top hit identified at the genus level was selected. Because of the lack of archaeal isolates, comparison against the NCBI nucleotide database, a larger database compared to other common databases (i.e., RDP and Greengenes), has been shown to provide more depth of information for archaeal sequences (Kan et al., 2011). The goal of the study was to compare over time and space and not make a definitive, taxonomic assignment for the sequences. Taxonomic assignments can be prone to mis-annotation with short, pyrosequence reads; therefore, a comparison between RDP and GenBank calls are provided in Supplementary Figure 2 (phylum-level) and Supplementary Figure 3 (genus-level) for the *Bacteria* datasets. Any sequences identified as the wrong domain were removed. Furthermore, any sequence with <80% identity or length of alignment to another SSU rRNA gene sequence was removed as a non-SSU rRNA gene sequence (Huse et al., 2010). In-house python scripts were used for sequence management and analysis.

### Data analysis

Rarefaction curves were generated on clusters at 97% similarity using the RDP Pyrosequencing Pipeline (Cole et al., 2009). Good's coverage was calculated for each sample as a measure of sampling completeness (Good, 1953). Chao1 species richness estimate and Shannon's diversity index were calculated on random subsamples to alleviate biases by sample size (Youssef and Elshahed, 2008; Dickie, 2010; Gihring et al., 2012). Briefly, 100 random samples were generated with replacement to a sample size of 1355 sequences for bacterial samples and 1175 sequences for archaeal samples. The average richness and diversity index were used for each sample. Hierarchical clustering and analysis of similarity (ANOSIM) were performed in R Statistical Software (v 3.0.0; R Foundation for Statistical Computing; Vienna, Austria) on Sorensen dissimilarity matrices.

## Results

Visible changes were not observed for the 63 and 75°C springs across time; however in 2009, the microbial mat in the 44°C spring (generally 1–2 cm in thickness) had torn throughout the spring and had curled up on itself to reveal the rock surface beneath (Figure 1). An increase in gas emission (via bubbling) or in water effluent was not observed and these tears were present upon return to the site in 2010. Temperature and pH were monitored *in situ* yearly and only slight variations were observed (Table 1). In 2009, a suite of aqueous geochemical analyses was performed on the 3 springs (Table 2). The following were not detected or were below quantifiable limit for all springs: Be, Cr, Fe, Co, Ag, Tl, Th, and U. The 63°C spring generally had lower concentrations of anions and trace elements, but was higher in DS, Ca, Mg, and Ba. In general, these springs showed elevated concentrations of Na and all of the anions tested (i.e., F^−^, Cl^−^, and SO^2−^_4_), indicative of a neutral-Cl spring.

**Table 1:** **Temperature and pH of the springs across 3 years**.

**Sample**	**Temp (°C)**	**pH**
44°C_2007	44	8.5
44°C_2008	38	8.1
44°C_2009	41	8.5
63°C_2007	63	8.5
63°C_2008	59	8.4
63°C_2009	63	8.5
75°C_2007	75	8.5
75°C_2008	74	8.5
75°C_2009	76	8.6

**Table 2:** **2009 Aqueous geochemistry**.

**Constituent^a^**	**Units**	**44°C_2009**	**63°C_2009**	**75°C_2009**
Conductivity	mS/cm	1.45	1.12	1.41
DS	mg/L	0.007	0.097	0.005
TC	mg C/L	52.8	37.2	38.4
NPOC	mg C/L	1.6	0.65	0.43
DIC (by difference)	mg C/L	51.2	36.5	38.0
TN	mg/L	0.33	0.11	0.16
F^−^	mM	10.2	7.9	9.7
Cl^−^	mM	45.5	34.8	42.2
SO^2−^_4_	mM	10.5	8.9	9.2
Na	mM	13.0	8.9	11.7
Mg	μM	Bql	17.9	Bql
Al	μM	1.7	1.7	1.7
K	μM	221	221	218
Ca	μM	46.5	161	42.9
V	μM	0.03	0.01	0.02
Mn	μM	0.09	0.21	0.12
Ni	μM	Nd	Nd	0.01
Cu	μM	Nd	Nd	0.18
Zn	μM	1.6	1.0	10.3
As	μM	11.2	5.2	10.7
Se	μM	0.05	0.05	0.05
Mo	μM	0.49	0.42	0.44
Cd	μM	0.01	0.01	0.01
Sb	μM	0.30	0.16	0.28
Ba	μM	0.08	0.22	0.06
Pb	μM	0.01	0.01	0.04

a Abbreviations: DS, dissolved sulfide, TC, total carbon, NPOC, non-purgable organic carbon, DIC, dissolved inorganic carbon, TN, total nitrogen, Nd, not detected, Bql, below quantifiable limit.

Pyrosequencing resulted in 83,465 high-quality sequences (31,842 bacterial sequences and 51,623 archaeal sequences). None of the PCR methods tested resulted in bacterial SSU rRNA gene amplification for the 75°C_2009 sample; however, archaeal SSU rRNA products were amplified and sequenced for this sample. Amplification of the bacterial community of this spring was consistently difficult, requiring more PCR cycles to yield product. When 40 cycles of PCR with 8 μL of 20× DNA concentrate proved unsuccessful in yielding DNA product for the 2009 sample, nested PCR was performed using universal bacterial primers FD1 and 1540R (Hwang et al., 2009) in the first round of PCR followed by PCR cleanup with the Wizard^®^ kit and then PCR with bacterial 530F and 100R primers as described above. A negative control was carried throughout the process and cycle numbers were varied for each round of PCR (5–10 cycles for the first round and 20–30 cycles for the second round). The ability to detect product in the sample was at or near the level of detection for background DNA present in the polymerase mix (recently reviewed in Mühl et al., 2010; Philipp et al., 2010). Nested PCR with 7 cycles in the first round and 27 cycles in the second consistently yielded product in the sample and no visible bands in the negative controls; however, upon sequencing, it was clear that the amplification was from the low-levels of contamination and not the sample as *Pseudomonas* and *Propionibacterium* were the dominant genera. Amplification and sequencing was repeated with similar results. Thus, bacterial community analysis for the 2009 75°C spring sample was not included in the analyses. Furthermore, the majority of forward sequences from the 75°C_2007 archaeal sample contained duplicate priming events (i.e., barcode-primer-barcode-primer-seq). For this reason, only the reverse sequences were used for analysis. Because the V6 region of the bacterial SSU rRNA gene has been shown to be problematic for identification and sample clustering recovery (Liu et al., 2007; Claesson et al., 2009; Claesson and O'Toole, 2010), these sequences (i.e., reverse sequences) were not included in the discussion and are not included in the sequence totals above. A comparison of the V4 and V6 region community compositions is shown in Supplementary Figure 1. However, little is known regarding short (250–300 bp) archaeal amplicons and identification; thus, both forward and reverse reads (roughly corresponding to the V5 and V6–V7 regions, respectively) were used for the archaeal sample analysis.

Rarefaction analysis of the bacterial samples indicated that the 63 and 75°C samples had reached near saturation while the 44°C spring samples contained more operational taxonomic units (OTUs = 97%) and had reached the curvilinear phase in the sampling effort (Supplementary Figure 4). Good's coverage calculations ranged from 0.865 to 0.999 corresponding to 7.4–1000 sequences required to identify a new OTU (Table 3). The lowest values were for the 44°C spring bacterial samples which were also the most diverse samples. Rarefaction analysis of the forward and reverse archaeal datasets differed in OTU predictions for the 44°C_2008 and 63°C_2009 samples, but both datasets predicted these samples to have the highest archaeal OTUs. Interestingly, these archaeal samples were not saturated in this sequencing effort (>5000 sequences for each sample) as shown by the rarefaction curves and Good's coverage (Table 3). OTU predictions for the remaining archaeal samples were similar for the forward and reverse datasets and Good's coverage suggested that the depth of sequencing was near saturation. The archaeal OTU predictions differed from year to year suggesting that estimated species richness was not static, and that population frequencies and distributions are dynamic despite relatively constant geophysicochemical conditions. However, there could also be changing environmental parameters that were not captured in the sampling.

**Table 3:** **Average diversity indices from 100 random subsets of 1355 sequences for *Bacteria* and 1175 sequences for *Archaea* and Good's Coverage**.

**Sample type**	**Sample**	**Sequences**	**OTUs (97%)**	**Chao1**	**Shannon's**	**Good's coverage**
Bacteria	44°C_2007	5988	1145	826	5.5	0.890
	44°C_2008	3604	827	687	5.1	0.865
	44°C_2009	5748	305	226	3.2	0.974
	63°C_2007	6033	944	694	4.9	0.904
	63°C_2008^a^	1355	246	423	4.3	0.911
	63°C_2009	4812	372	296	4.0	0.967
	75°C_2007	2164	172	159	3.0	0.954
	75°C_2008	2138	36	34	2.1	0.993
Archaea forward	44°C_2007^a^	252	45	66	2.7	0.921
	44°C_2008	5232	618	470	4.1	0.941
	44°C_2009	2648	101	89	1.9	0.980
	63°C_2007	1483	126	119	2.6	0.960
	63°C_2008^a^	1175	99	97	2.7	0.955
	63°C_2009	8490	652	439	3.9	0.968
	75°C_2008	3668	79	68	2.2	0.994
	75°C_2009	4930	26	20	0.6	0.998
Archaea reverse	44°C_2007	1226	68	66	2.6	0.981
	44°C_2008	1772	268	249	3.9	0.925
	44°C_2009^a^	826	50	71	1.7	0.972
	63°C_2007	4675	233	180	2.8	0.975
	63°C_2008	4111	233	183	3.4	0.970
	63°C_2009	2245	230	213	3.6	0.955
	75°C_2007	3033	28	24	1.1	0.997
	75°C_2008	2441	88	81	2.3	0.984
	75°C_2009	3416	16	13	1.5	0.999

a Richness and diversity indices were calculated on the complete dataset. No subset was taken.

Chao1 species richness and Shannon's diversity index were calculated on random subsets for each sample (1355 sequences for *Bacteria* and 1175 sequences for *Archaea*). The averages of these subsets are provided in Table 3. Bacterial species richness and diversity was consistently lower in the 75°C spring and decreased across time in all springs similar to the results observed in the rarefaction analyses. Archaeal richness and diversity was more variable across time, but was consistently lowest in the 75°C spring.

**Figure 2:**
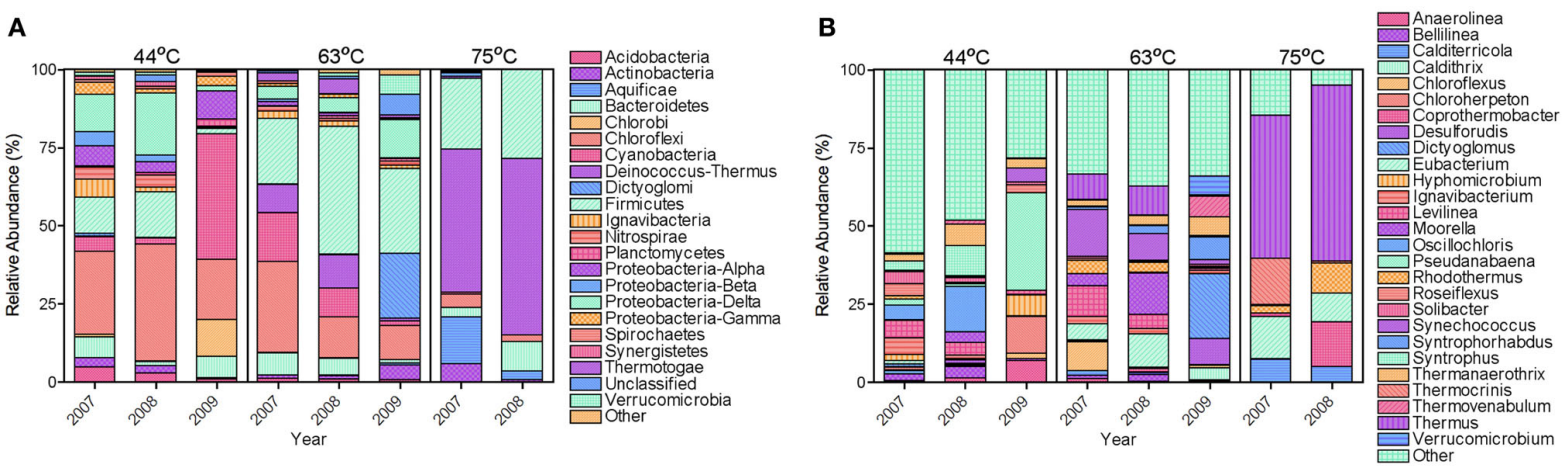
**Relative abundance of bacterial phyla (A) and genera (B).** Phyla **(A)** and genera **(B)** with a relative abundance <1 and <4%, respectively, were grouped as Other.

The bacterial populations were similar across time on the phylum and genus level for the 63 and 75°C spring samples, but differed in the 44°C spring samples (Figure 2). The 75°C spring was ~50% *Thermus* and the remaining 50% comprised of thermophilic genera. The 63°C spring was predominately *Firmicutes* and *Chloroflexi*. While 2007 and 2008 samples were also dominant in *Cyanobacteria* and *Deinococcus-Thermus*, the 2009 sample showed a decrease in these phyla and an increase in *Dictyoglomi*. On the genus level, the 2007 and 2008 samples for the 63°C spring differed in the relative abundance of *Chloroflexus*. The 2009 sample showed an increase in relative abundance of *Dictyoglomus, Desulforudis, Thermovenabulum*, and *Verrucomicrobium* compared to previous years. The 2007 and 2008 samples for the 44°C spring were similar on the phylum level and *Chloroflexi* were predominant (Figure 2). These samples were very diverse with genera <4% relative abundance comprising >50% of the total relative abundance. *Levilinea* was the genus with highest abundance and was only 5.6% of the total relative abundance. The 2008 sample showed a slight predominance of *Oscillochloris* and *Syntrophus* (14 and 10%, respectively) compared to other genera. The 2009 sample was less diverse and demonstrated a shift in dominance to *Cyanobacteria*, predominantly from the *Pseudanabaena* genus. Cluster analysis of the annual samples with the bacterial dataset resulted in distinct clustering by spring with the 75°C spring samples being quite dissimilar to samples from the other springs (Figure 4A).

**Figure 3:**
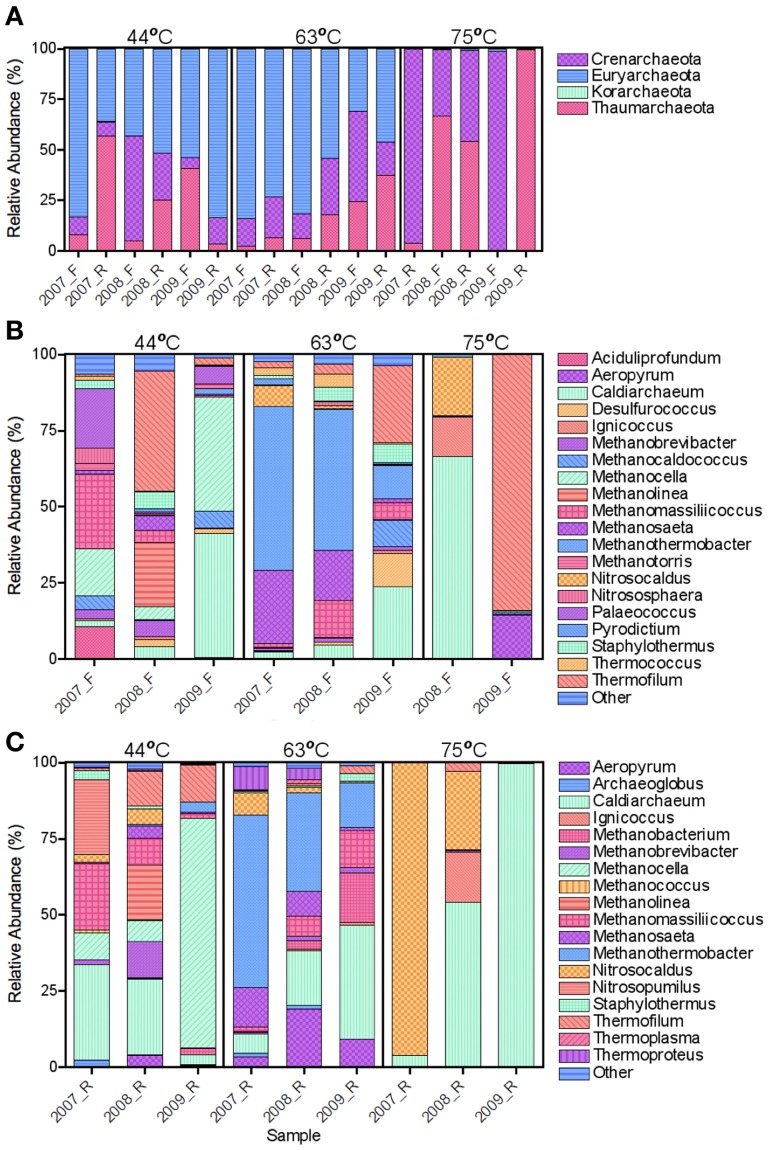
**Relative abundance of archaeal phyla (A) and forward (B) and reverse (C) genera.** Forward **(B)** and Reverse **(C)** genera with a relative abundance <2 and <1%, respectively, were grouped as Other. *Korarchaeota* were observed at low relative abundances (<0.4%) in the 44°C_2007 and 63°C_2007 reverse datasets.

In general, the archaeal populations fluctuated more across time compared to the bacterial populations (Figure 3). Similar to the bacterial samples, many archaeal genera shared dominance (i.e., more even population distribution) in the 44°C spring. *Methanomassiliicoccus* and *Methanocella* were dominant in both the forward and reverse datasets in 2007 and *Thermofilum* and *Methanolinea* were dominant in both datasets in 2008. The remaining predominant genera varied by SSU rRNA gene region. *Methanocella* was dominant for both SSU rRNA gene datasets in 2009, but shared dominance with *Caldiarchaeum* in the forward sequence set.

The 2007 and 2008 63°C spring showed the highest consistency across time and SSU rRNA gene region of archaeal relative abundances with the dominance of *Methanothermobacter* and *Methanosaeta* (Figures 3B,C). Though the 2009 sample differed in relative abundances of these genera, methanogens remained predominant regardless of SSU rRNA gene region. *Korarchaeota* were detected in 44°C_2007 and 63°C_2007 reverse datasets, but in low abundances (0.3 and 0.06% relative abundances, respectively). Each of the 3 OTUs that comprised the *Korarchaeota* group were most similar to the candidate genus *Korarchaeum* with a percent similarity of only 82%. At the time of manuscript completion, this was the only member of the *Korarchaeota* phylum in the NCBI nucleotide database. *Nanoarchaeota* sequences were not detected, though the primers have been shown to amplify this phylum (Baker et al., 2003).

**Figure 4:**
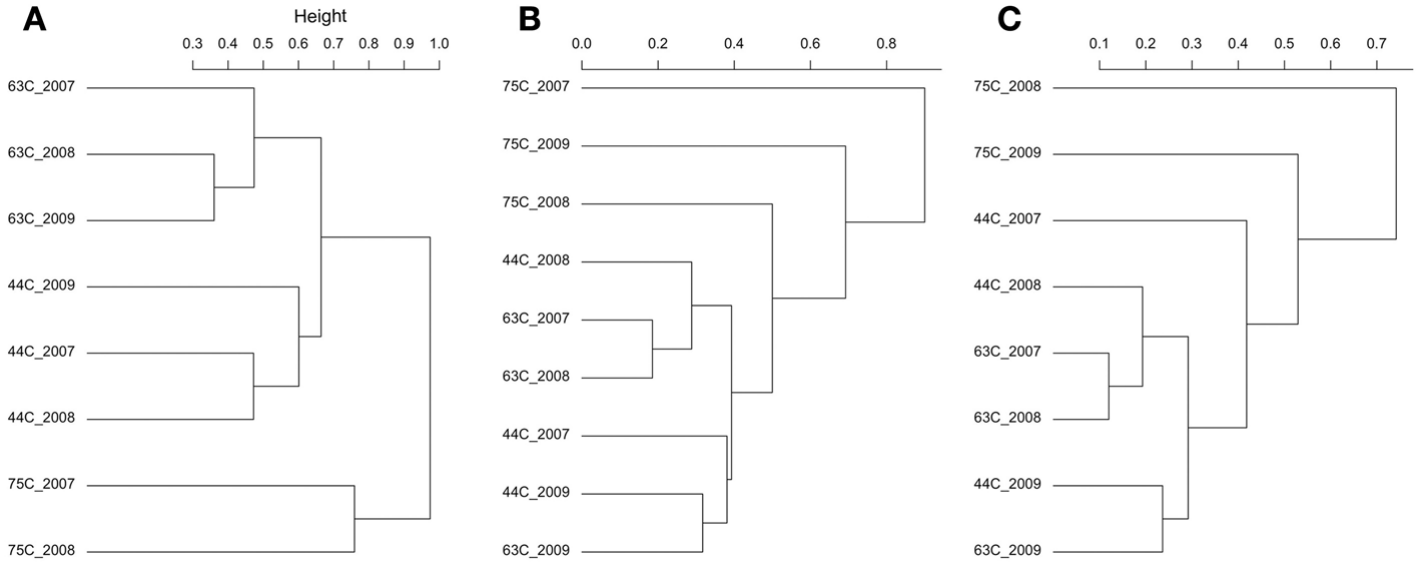
**Hierarchical clustering of the springs based on bacterial (A), archaeal forward (B), and archaeal reverse (C) communities.** Sorenson dissimilarity matrices were used to cluster samples.

The 75°C spring demonstrated large shifts in the archaeal populations with results differing by SSU rRNA gene region (Figure 3). Only the reverse sequences were used for the 2007 sample (above) and were predominantly *Nitrosocaldus*. Though still dominant in 2008, it was secondary in relative abundance to *Caldiarchaeum*. The 2008 sample showed similar relative abundances in both SSU rRNA gene regions. The 2009 sample was starkly different depending on gene region. The forward sequences indicated an abundance >75% for *Thermofilum* (accession no. GU187356), and the majority of remaining sequences were *Methanosaeta*. Both *Thermofilum* and *Methanosaeta* are *Crenarchaeota*. However, the reverse sequences indicated a predominance (99%) of *Caldiarchaeum*, a *Thaumarchaeota* (accession no. JN881573). Representative sequences had ~90% identity to either genus; thus the identification is difficult with the limited diversity of currently available sequences. Targeted isolations and sequencing will be needed to elucidate the identity of these putative populations. Cluster analysis of the annual samples with the archaeal dataset resulted in distinct clustering by the 75°C spring samples that were distinct from each other and the other wells (Figures 4B,C). In some instances, the 44 and 63°C samples were clustered (the 44°C_2009 sample clustered with the 63°C_2009 sample). ANOSIM confirmed that the spring from which the samples originated was a significant variable in sample clustering (*P*-value = 0.005, 0.016, 0.003 for the bacterial, archaeal forward, and archaeal reverse datasets, respectively).

**Figure 5:**
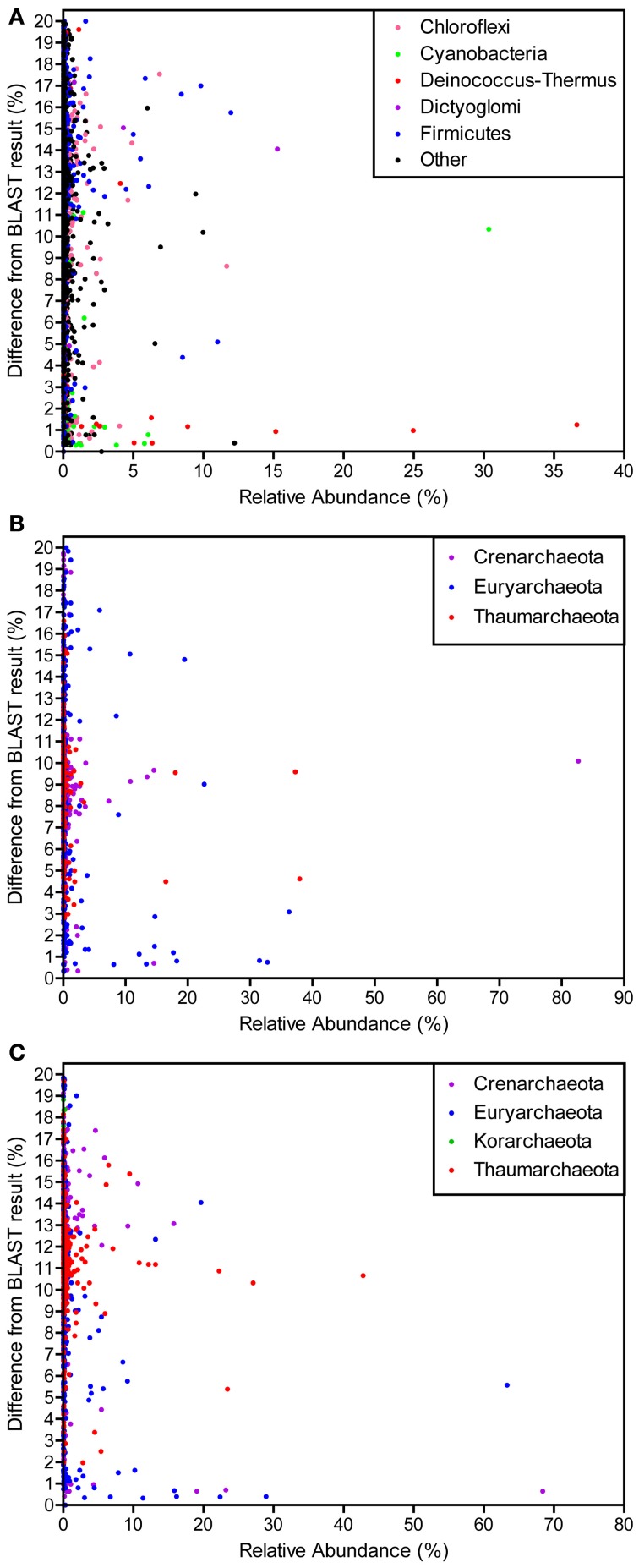
**Difference from BLAST result for OTUs of varying relative abundances for the bacterial (A), archaeal forward (B), and archaeal reverse (C) datasets.** The relative abundance of each OTU (clustered at 97% similarity) is plotted against the percent difference from the most similar BLAST result identified at the genus level.

Many OTUs were different from any known organisms in the NCBI nucleotide database (Figure 5). The majority of OTUs with high similarity to a known organism were closely related to *Deinococcus-Thermus* and *Cyanobacteria*. Those at low relative abundance varied from 0 to 20% different from any known organisms, the point at which sequences were no longer considered as SSU rRNA gene sequences. The archaeal datasets (i.e., forward and reverse) had similar results in that relatively few OTUs were similar to anything in the database, even for those high in abundance.

## Discussion

Due to the remote nature of the HLGB, little is known regarding the microbial communities of these thermoalkaline springs. We characterized the bacterial and archaeal communities of three alkaline springs with similar geochemistry but differing in temperature, and the springs demonstrated a decrease in diversity upon increasing temperature. Though many of the OTUs were dissimilar (>10% different) from known organisms, these taxonomic comparisons facilitated sample-to-sample comparisons as well as allowed for basic physiologic presumptions that were further supported by the spring characteristics. The bacteria datasets demonstrated a general shift from *Cyanobacteria* and *Chloroflexi* at 44°C to the observation of *Deinococcus-Thermus* at 63°C and, finally, a predominantly *Deinococcus-Thermus* population at 75°C. The results demonstrated a transition from a moderately thermophilic, photosynthetic population to a mixed photosynthetic and thermophilic population and, finally, to a non-photosynthetic thermophilic population. These results were supported by the mat characteristics in the spring, which shifted with increasing temperature from a red, *Chloroflexi* mat to a green cyanobacterial mat and finally no visible mat or pigments characteristic of photosynthesis. The upper temperature limit for prokaryotic photosynthesis is considered to be 73–75°C (Brock, 1967; Hamilton et al., 2012). With the exclusion of photosynthetic organisms by temperature, *Thermus* predominance in the 75°C spring was not surprising. *Thermus* spp. are common in neutral and alkaline springs in YNP and generally have an optimum temperature between 70 and 75°C (da Costa et al., 2006). These community differences suggested that temperature was primarily driving the community composition.

Relatively few community studies in neutral and alkaline springs in YNP have included *Archaea*, which have been considered to be rare in these springs (Reysenbach et al., 1994; Hugenholtz et al., 1998; Inskeep et al., 2010). Yet, membrane lipid surveys have shown ubiquity and co-occurrence of *Bacteria* and *Archaea* throughout YNP (Schouten et al., 2007). Our results demonstrated a diverse archaeal community that varied with temperature. The 44 and 63°C springs comprised of predominately acetoclastic and hydrogenoclastic methanogen populations, but the 75°C spring was predominately thermophilic *Archaea*. Methanogenesis has been shown to be inhibited at temperatures above 65–70°C in other neutral and alkaline springs in YNP (Ward, 1978; Zeikus et al., 1980), although some methanogens can grow at temperatures >75°C. Further work is needed to understand the biotic and abiotic parameters that impact methanogens at higher temperatures.

The 75°C spring populations differed across time and by SSU rRNA gene region. In 2007, the reverse library indicated a >90% relative abundance of *Candidatus Nitrosocaldus*, an ammonia-oxidizing crenarchaeote originally detected in Heart Lake enrichments from a 72°C spring (de la Torre et al., 2008) and the dominant population in neutral and alkaline hydrothermal vents in Yellowstone Lake (Kan et al., 2011). It is not known if members of this genus were active in our 75°C spring as nitrite production ceased at 74°C in the aforementioned HLGB enrichments. Interestingly, the *Candidatus Nitrosocaldus* was not detected in the 2009 sample, suggesting that it may be transient in this spring. The 2009 75°C spring samples were almost exclusively *Thermofilum* or *Candidatus Caldiarchaeum*, depending on SSU rRNA gene region. *Thermofilum* is a sulfur-respiring crenarchaeote that is generally considered a moderate acidophile (Huber et al., 2006). The genome of *Candidatus Caldiarchaeum* was obtained from a metagenome of geothermal waters in a subsurface mine, but it has not been isolated or characterized (Nunoura et al., 2011). Whether this genus is a deeply rooted member of the *Thaumarchaeota* or a new phylum (proposed as *Aigarchaeota*) is still debated (Brochier-Armanet et al., 2011; Pester et al., 2011). Representative sequences from the 2009 75°C spring libraries were only ~90% similar to either of these genera and the difference in identity between the forward and reverse libraries may be a consequence of low identity. The use of short amplicon sequencing via high-throughput technologies is becoming common; yet, the majority of method development has been performed on bacterial sequences. Methods development on archaeal SSU rRNA gene sequences warrants further inquiry in order to achieve better resolution.

In 2009, geochemical analysis was performed and demonstrated minimal differences across the springs. When slight differences were observed (i.e., such as in Ca^2+^, Mg^2+^, and dissolved sulfide), the difference was in the 63°C spring and did not follow a trend that would suggest that it was driving the community differences such as in the photosynthetic or methanogenic populations. An extensive geochemical survey of the HLGB was performed in 2009 by Lowenstern et al. (2012), but did not include springs in the western portion of the lower basin. Our geochemistry compliments this study with an additional region of the basin and corresponds with the trends observed throughout alkaline springs in the HLGB.

At an alkaline pH of ~8.5 and little differences in aqueous geochemistry, springs with varying temperatures from 44 to 63 to 75°C had major differences in both bacterial and archaeal populations, namely in the presence of photosynthetic bacteria and methanogens in the lower temperature springs and their absence in the 75°C spring. Cluster analysis of samples and ANOSIM demonstrated that the spring from which the sample originated was significant. Many variables could be contributing to the differences in spring; however, temperature was the only parameter measured that showed large differences between springs. NPOC was the only other geochemical measurement that corresponded to the temperature changes (decreased with increasing temperature), though the changes between springs were small. The 63°C spring was generally lower in heavy metals and anions than the other springs. This may be due to this spring being a mixture of stream water (coming from springs upstream) and source water, causing a dilution of the constituents measured. The 44 and 63°C springs tended to be the most geochemically different springs when the three springs were compared across the measured geochemistry; however, these springs were more similar to each other than the 75°C spring based upon communities. These results suggest temperature as a primary driver in community differences and coincide with previous work that has shown chlorophyll-driven phototrophy is limited to <70°C (Brock, 1967; Hamilton et al., 2012).

Community variation in both bacterial and archaeal populations across time were observed, and the most variance was observed in the 75°C spring. Interestingly, the 75°C spring is the most isolated (e.g., surface hydrological connectivity was not observed) yet displayed the most temporal variability over the three tested years. In addition, the other two springs had observable connections to run-off streams and/or wetlands, and these features could contribute to some of the temporal variability (or vice versa contribute to stability). Spring run-off may alter the surface connectivity of these springs, but spring access to the area is severely limited due to grizzly bear activity. The temporal variation may be due to unknown geochemical parameters contributing to temporal community dynamics, and/or shifts in respective populations that are driven by specific, co-dependent interactions as temperature varied little temporally. In addition, the temporal variation may be isolated to the bulk phase or solid phase (e.g., sediment or mat), and further characterization will be needed to delineate population dynamics across time and space. Further community and geochemical comparisons of these springs and other springs in the HLGB warrant further inquiry and would better clarify the geochemical factors driving community composition as well as invasion/stability dynamics.

An analysis of the similarity of each OTU to the closest representative in the NCBI nucleotide database identified at the genus level indicated that the majority of OTUs were not similar (≥10% different) to any known genus (Figure 5). In fact, relatively few were <3% different from another organism. Many dominant OTUs were ≥10% different than any known genus and could represent groups with unknown physiologies. Bacterial sequences that were similar to a known organism were generally *Deinococcus*-*Thermus* and *Cyanobacteria*, two groups extensively studied and with isolates from YNP (reviewed in Ward et al., 1998; da Costa et al., 2006). Both SSU rRNA gene regions analyzed for archaeal libraries demonstrated similar results and few OTUs were similar to known genera. Those that were similar to a known organism were exclusively *Crenarchaeota* and *Euryarchaeota*. The majority of *Thaumarchaeota* clustered at ~10% different from any known organisms and *Korarchaeota*, with only 3 OTUs and exclusively in the reverse dataset, were 18–19% different than the only *Korarchaeota* in the database, *Candidatus Korarchaeum*. These results indicate the presence of potentially new phylogenetic groups in both the archaeal and bacterial communities. With the aid of this community analysis as a guideline, large culturing and sequencing efforts in the HLGB merit investigation.

### Conflict of interest statement

The authors declare that the research was conducted in the absence of any commercial or financial relationships that could be construed as a potential conflict of interest.
