# Supplementary material for: Archaeal and bacterial communities in three alkaline hot springs in Heart Lake Geyser Basin, Yellowstone National Park
Source: Front Microbiol. 2013 Nov 12;4:330. doi: 10.3389/fmicb.2013.00330 (PMC3824361; doi:10.3389/fmicb.2013.00330)
Supplement: Supplementary file 1 [file DataSheet1.PDF]

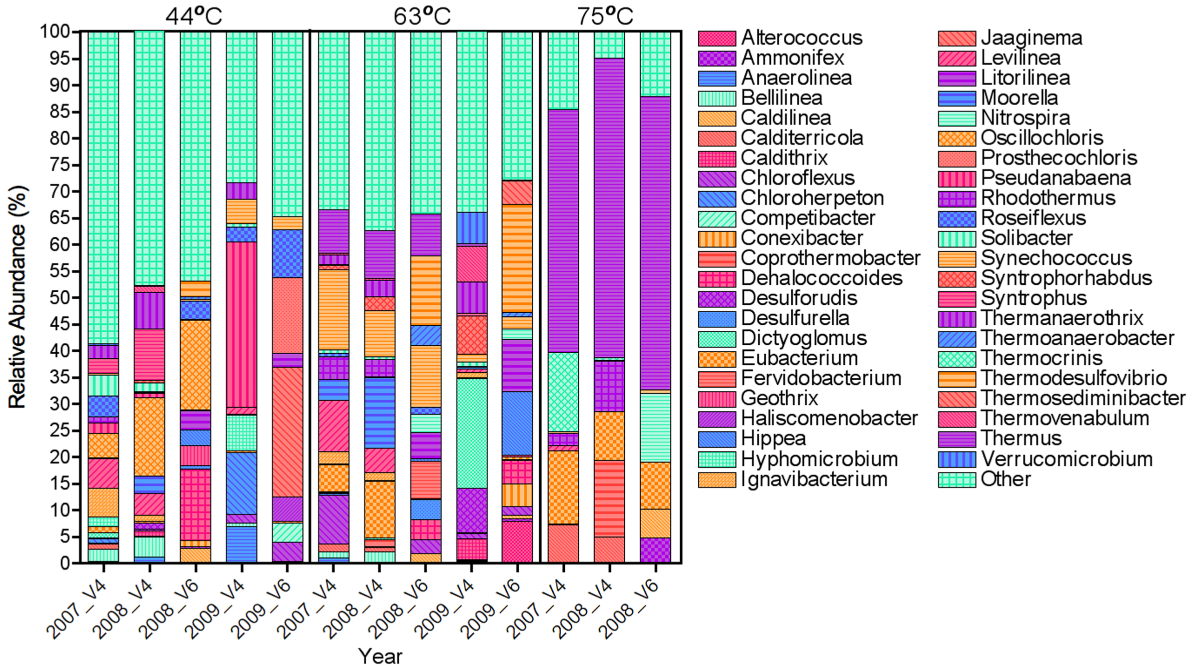

Supplementary Figure 1. Relative abundance of bacterial genera comparing the V4 and V6 regions of the SSU rRNA gene. Genera with a relative abundance <4% were grouped as Other.

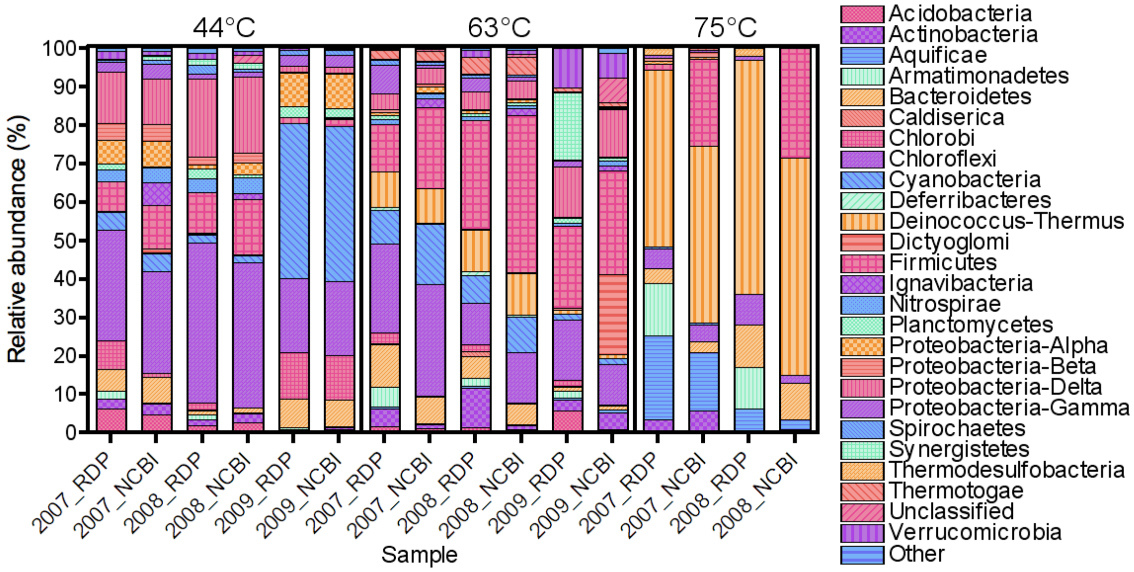

Supplementary Figure 2. RDP and NCBI Blast comparison at the phylum level. The bacterial V4 dataset was used to compare RDP and NCBI identifications on the phylum level. Phyla <1% relative abundance in all samples were grouped as Other.

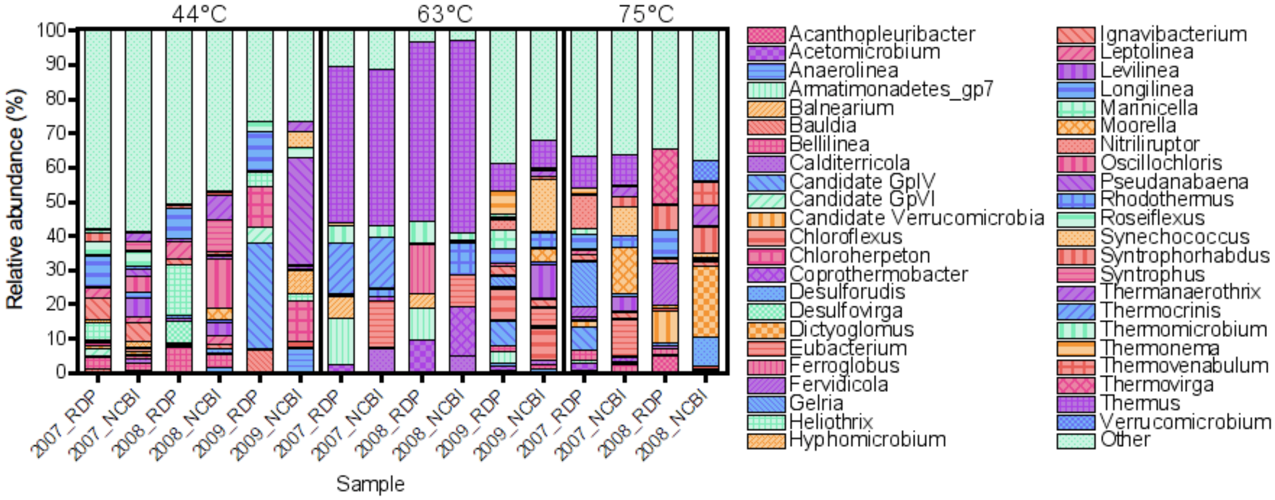

Supplementary Figure 3. RDP and NCBI Blast comparison at the genus level. The bacterial V4 dataset was used to compare genus-level identifications from RDP and NCBI. Genera <4% relative abundance in all samples were grouped as Other.

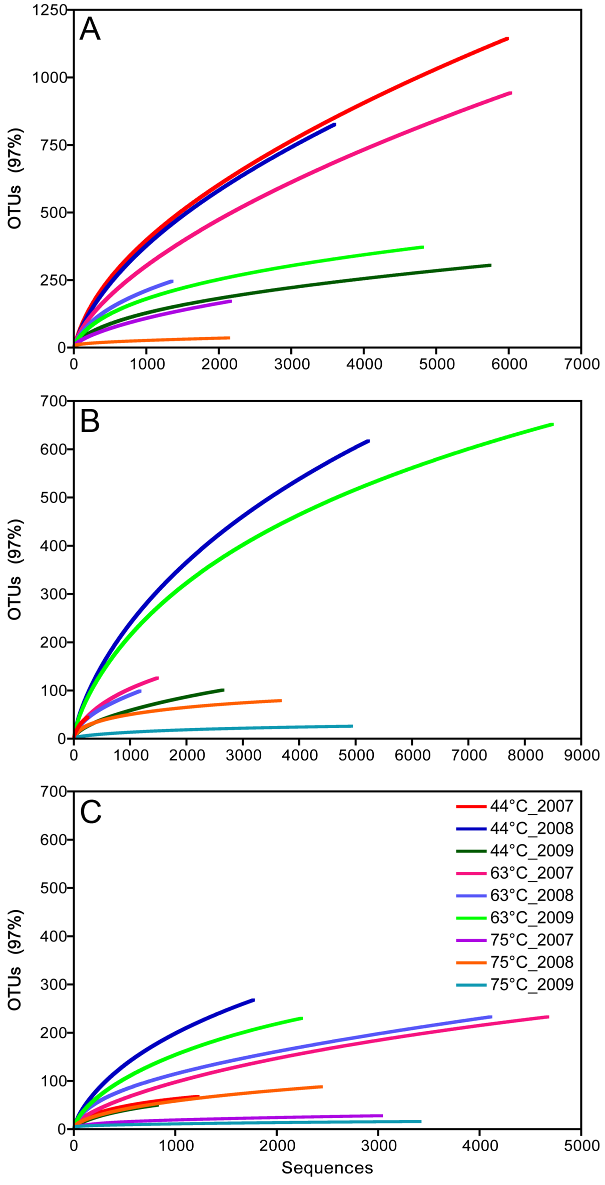

Supplementary Figure 4. Rarefaction curves of bacterial (A), archaeal forward (B), and archaeal reverse (C) datasets. Operational taxonomic units (OTUs) were complete-linkage clustered at 97% similarity.
